# Advancing acute respiratory failure management through artificial intelligence: a call for thematic collection contributions

**DOI:** 10.1186/s40635-024-00629-4

**Published:** 2024-05-07

**Authors:** Zhongheng Zhang, Jakob Wittenstein

**Affiliations:** 1 Department of Emergency Medicine, Sir Run Run Shaw Hospital, Zhejiang University School of Medicine, Hangzhou, Zhejiang China; 2 Department of Anesthesiology and Intensive Care Medicine, Pulmonary Engineering Group, University Hospital Carl Gustav Carus Dresden, TUD Dresden University of Technology, Dresden, Germany

In recent years, artificial intelligence (AI) has permeated virtually every facet of modern life, revolutionizing industries from finance to transportation, and now, healthcare [1]. The COVID-19 pandemic has starkly illuminated the vulnerability of our healthcare systems when faced with sudden surges in critically ill patients, particularly those with respiratory complications. The need for rapid, accurate diagnosis and personalized treatment plans has never been more pressing [2, 3]. AI offers a unique opportunity to analyze vast amounts of data with unprecedented speed and accuracy, which can lead to breakthroughs in early detection, prevention, and treatment strategies for ARF [4]. ARF patients are usually treated with mechanical ventilation in the intensive care unit and parameters of mechanical ventilation are continuous (validated every minute) and hence the dynamics of MV provide a large amount of data which are difficult to analyze without AI.

The motivation to launch this thematic collection is rooted in the recognition of the potential power of AI, which, when integrated with clinical expertise, can significantly enhance patient care. By leveraging AI's predictive analytics, we can improve clinical decision-making, optimize resource allocation, and develop more targeted interventions. Moreover, AI's ability to process complex imaging and physiological data can facilitate a deeper understanding of the pathophysiological mechanisms underlying acute respiratory failure, paving the way for novel therapeutic approaches [5, 6].

In this context, the collection aims to bring together the latest research and insights at the intersection of AI and ARF management. We seek to highlight the innovative applications of AI that can transform the way we approach this critical condition, from the initial triage to the long-term management of complications. By showcasing the potential of AI to revolutionize the management of ARF, such as risk stratification, subphenotype identification and individualized treatment, we hope to galvanize the healthcare providers in critical care setting to adopt these cutting-edge technologies, ultimately leading to improved ARF outcomes and a more resilient healthcare system in the face of future challenges.

The scope of this thematic collection is broad and ambitious. We welcome (1) original researches that delves into the development and validation of AI algorithms for early prediction, diagnosis, risk stratification, prognosis assessment, and treatment of ARF; (2) studies that demonstrate the integration of AI-driven decision support systems into clinical practice, optimizing mechanical ventilation strategies and patient monitoring; (3) submissions that explore novel data sources such as imaging and multi-omics data and AI-driven analytics for continuous monitoring, prediction, and management of acute respiratory failure exacerbations; (4) researches addressing ethical considerations, challenges, and opportunities associated with the adoption of AI technologies in acute respiratory failure management.

The research published in this collection will have a profound impact on patient outcomes, enabling earlier detection, more accurate diagnosis, and optimized treatment strategies. We are committed to fostering a dialogue that bridges the gap between AI research and clinical application, ensuring that the latest advancements benefit those who need it most. We invite researchers, clinicians, and industry professionals to join us in shaping the future of acute respiratory failure management.

## Data Availability

Not applicable.
